# Factors Associated with Overutilization of Computed Tomography of the Cervical Spine

**DOI:** 10.5811/westjem.58948

**Published:** 2023-08-28

**Authors:** Karl T. Chamberlin, Maureen M. Canellas, Martin A. Reznek, Kevin A. Kotkowski

**Affiliations:** University of Massachusetts Chan Medical School, Department of Emergency Medicine, Worcester, Massachusetts

## Abstract

**Introduction:**

Despite the wide availability of clinical decision rules for imaging of the cervical spine after a traumatic injury (eg, NEXUS C-spine rule and Canadian C-spine rule), there is significant overutilization of computed tomography (CT) imaging in patients who are deemed to be at low risk for a clinically significant cervical spine injury by these clinical decision rules. The purpose of this study was to identify the major factors associated with the overuse of CT cervical spine imaging using a logistic regression model.

**Methods:**

This was a retrospective review of all adult patients who underwent CT cervical spine imaging for evaluation of a traumatic injury at a tertiary academic emergency department (ED) and three affiliate community EDs in January and February 2019. We performed multivariable logistic regression to identify factors associated with obtaining CT cervical spine imaging despite low-risk classification by the NEXUS C-spine Rule.

**Results:**

A total of 1,051 patients underwent CT cervical spine imaging for traumatic indications during the study period, and 889 patients were included in the analysis. Of these patients, 376 (42.3%) were negative by the NEXUS C-spine rule. Variables that were associated with increased likelihood of unnecessary imaging included age over 65, Emergency Severity Index (ESI) score 2 and 3, arrival as a walk-in, and anticoagulation status. Patients who presented to the tertiary academic ED had a significantly lower likelihood of unnecessary imaging. Twenty-one patients (2.4%) were found to have cervical spine fractures on imaging, two of whom were negative by the NEXUS C-spine rule, but neither had a clinically significant fracture.

**Conclusion:**

Cervical spine imaging is vastly overused in patients presenting to the ED with traumatic injuries, as adjudicated using the NEXUS C-spine rule as a reference standard. Older age, ESI level, arrival as a walk-in, and taking anticoagulation drugs were associated with overutilization of CT imaging. Conversely, presenting to the tertiary academic ED was associated with a lower likelihood of undergoing unnecessary imaging. This model can guide future interventions to optimize ED CT utilization and minimize unnecessary testing.

Population Health Research Capsule What do we already know about this issue?

*CT of the cervical spine is frequently performed on patients who are low risk for a clinically significant injury by well-validated criteria like the NEXUS C-spine rule.*

What was the research question?

*We sought to identify factors that are associated with overutilization of CT cervical spine.*

What was the major finding of the study?

*Variables associated with overuse include age > 65, lower Emergency Severity Index score, arrival not by EMS, anticoagulation, and non-university academic site.*

How does this improve population health?

*These variables may guide future interventions to reduce overuse of CT cervical spine, resulting in improved ED efficiency, reduction in radiation exposure, and lower healthcare costs.*

## INTRODUCTION

Evaluation of potential cervical spine injury is a common reason for presentation to the emergency department (ED). Annually, more than 2.5 million patients in the United States seek care at an ED for evaluation of a potential injury to the cervical spine.^1^ It has been previously estimated that 3–10% of these patients may have clinically significant cervical spine injuries.^2^^–^^5^ In recent decades, the volume of radiographic imaging performed in EDs has increased exponentially, particularly computed tomography (CT).^6^^,^^7^ This dramatic increase in imaging studies presents numerous potential negative implications, including increased healthcare costs, risks of radiation, longer lengths of stay, more incidental findings, and inefficiencies in ED throughput.^8^^–^^10^ Therefore, it is a valuable objective to moderate the use of imaging in the ED to avoid unnecessary imaging.

Clinical decision rules have gained traction in emergency medicine to assist with decision-making, and several clinical decision rules have been well-studied and validated to determine the need for imaging of the cervical spine after a traumatic injury. The most used decision rules are the NEXUS C-spine rule and the Canadian C-spine rule.^11^^,^^12^ The NEXUS C-spine rule establishes criteria that can be used to risk-stratify patients and thereby identify patients who are at low risk for a clinically significant cervical spine injury and for whom cervical spine imaging is thus unnecessary.

Although these clinical decision rules are widely available, utilization of these rules is variable. Prior literature has demonstrated that approximately 25% of CT C-spine studies were performed on patients who did not meet NEXUS criteria for imaging.^13^^–^^16^ To date it is not well understood why the high rate of CT C-spine overutilization persists despite validated decision rules having been in place and broadly communicated for the last two decades. Understanding factors contributing to the overuse of CT C-spine may inform targeted strategies to increase decision-rule adherence and thereby reduce unnecessary imaging studies. Our objective in this study was to identify the major factors associated with the overutilization of CT cervical spine imaging, as adjudicated by the NEXUS C-spine rule as a reference standard, using a logistic regression model.

## METHODS

This was a multicenter, retrospective review of all adult patients who underwent CT cervical spine imaging for evaluation of blunt traumatic spinal injury. We obtained data on patients who presented to an urban, tertiary academic ED (approximately 77,000 annual patient encounters) and three affiliate community EDs (ranging from approximately 13,000–43,000 annual patient encounters) in January and February 2019. Exclusion criteria included patients <18 years of age, and CT indications that were nontraumatic, penetrating trauma, or unspecified. This study was granted an institutional review board exemption.

We extracted data from the electronic health record on all patients who underwent CT of the cervical spine in the ED during the pre-specified period. In addition to the demographic data obtained, each chart was manually reviewed by a single reviewer for the presence of each of the five NEXUS criteria: focal neurological deficit; midline cervical spine tenderness; distracting injury; intoxication; or altered mental status. During initial chart review, we also collected data on the Canadian criteria. However, there was inadequate data to proceed with analysis of the Canadian C-spine rule because of insufficient clinical documentation of low-risk factors and range of motion of the neck. Due to the demands of chart review and the number of cases, we selected a two-month study period for feasibility.

We defined the NEXUS criteria as follows: 1) focal neurological deficit—any acute abnormality of the motor or sensory exam that was not attributed to pain; 2) midline tenderness—bony or midline tenderness of the neck or cervical spine; 3) distracting injuries—fractures of the humerus, radius, ulna, femur, tibia, fibula, or sternum, multiple rib fractures, or any other injury that was documented as “distracting”; 4) intoxication—clinical signs of intoxication on exam, history of recent alcohol intake, or a detectable serum ethanol level; and 5) altered mental status—Glasgow Coma Score between 3–14 as documented by the physician or nurse, or documentation that the patient was disoriented, confused, nonverbal, or unresponsive. Each of these criteria has high clinical relevance that warrants documentation in the health record; therefore, NEXUS criteria that were not documented were presumed to be absent. Patients who were negative for all five criteria were deemed at low risk for a clinically significant cervical spine injury by NEXUS criteria. We classified the CT cervical spine studies as “overutilization” if they were ordered on patients who were at low risk by NEXUS criteria.

We performed multivariable logistic regression to identify factors associated with obtaining CT cervical spine imaging despite low-risk classification by the NEXUS C-spine rule. The following variables were included in the regression analysis: age; gender, mechanism of injury; ED site; Emergency Severity Index (ESI) score; arrival time of day; arrival day of week; means of arrival; anticoagulation status; concurrent head CT; trauma activation level (ie, 1, 2, 3, or none); physician level of training (ie, resident or attending); and ordering attending. There were no missing or imputed values. Trauma activation level, ESI 1, physician level of training, and ordering attending were removed due to significant multicollinearity (over 70%) with ED site. Variable statistical significance was denoted by a *P*-value of less than 0.05. We performed all analyses using R version 4.0.2 (R Foundation for Statistical Computing, Vienna, Austria).

## RESULTS

**Figure 1.**
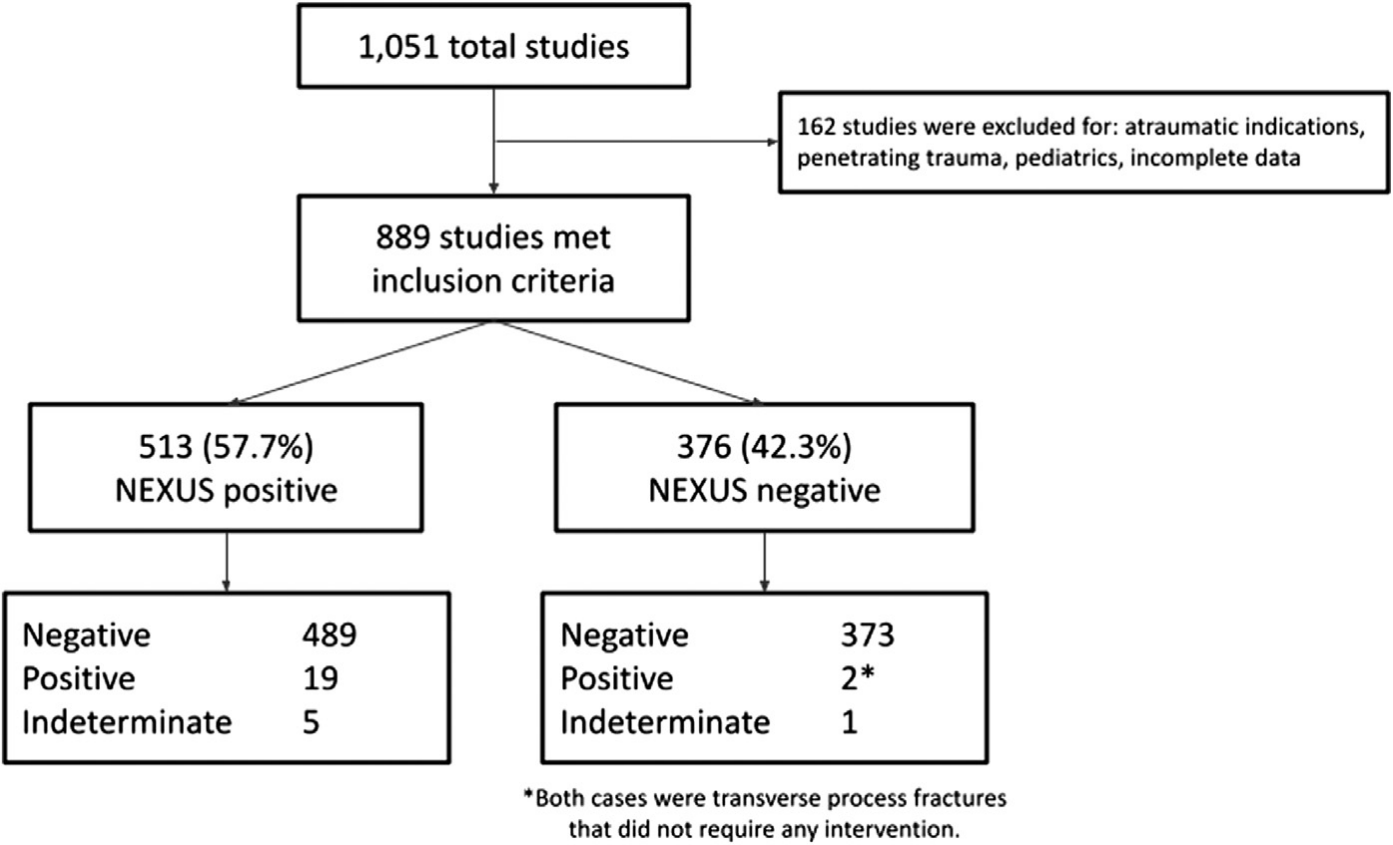
Of the 1,051 computed tomography C-spine studies performed in the study period, 889 met inclusion criteria. Forty-two percent of the studies were performed on NEXUS-negative patients, and none of the NEXUS-negative patients were found to have a clinically significant cervical spine injury.

During the two-month study period, 1,051 CTs of the cervical spine were performed across all sites, and 889 met the inclusion criteria. Baseline characteristics of the study population are shown in Table 1. Of the scans that met inclusion criteria, 376 (42.3%) did not meet any of the NEXUS criteria. Notably, of the 376 CTs that were performed on NEXUS-negative patients, 373 (99.2%) were negative for any acute fracture. Two images were positive for clinically insignificant transverse process fractures that did not require intervention. One was nondiagnostic due to motion artifact, and this patient did not subsequently require further imaging, intervention, or management. None of the patients in the NEXUS-negative group were identified to have a clinically significant cervical spine fracture. In the NEXUS-positive group, 19/513 CTs (3.7%) were positive for fracture, and five were indeterminate for fracture. All five patients with indeterminate initial imaging underwent follow-up imaging with MRI or repeat CT. Four had no injury on follow-up imaging, and one was found to have a clinically significant fracture. These results are shown in Figure 1.

**Table 1.** Population baseline characteristics for adult patients who presented to four emergency departments for evaluation of potential cervical spine traumatic injury.

Variable	n	%
Age		
18–44	175	19.7
45–64	207	23.3
65–84	300	33.7
≥85	207	23.3
Gender		
Male	438	49.3
Female	451	50.7
Mechanism of injury		
Fall	660	74.2
MVC	154	17.3
Assault	20	2.2
Seizure	17	1.9
Pedestrian struck	10	1.1
Other	28	3.1
Site of arrival		
Academic ED	585	65.8
Community ED #1	95	10.7
Community ED #2	172	19.3
Community ED #3	36	4.0
Emergency Severity Index score		
1	52	5.8
2	294	22.1
3	431	48.5
4	105	11.8
5	1	0.1
Trauma activation level		
1	24	2.7
2	169	19.0
3	17	1.9
None	679	76.4
Means of arrival		
Emergency medical services	728	81.9
Walk-in	161	18.1
Anticoagulation		
Yes	135	15.2
No	748	84.1
Concurrent CT head		
Yes	775	87.2
No	114	12.8
Role of ordering clinician		
Resident	480	54.0
Attending	404	45.4
Advanced practice practitioner	4	0.4

*MVC,* motor vehicle collision; *ED,* emergency department; *CT*, computed tomography.

The regression analysis output is shown in Table 2. Variables associated with increased likelihood of unnecessary imaging include age 65–84 (*P* < 0.001); age ≥85 years (*P* < 0.001); arrival by walk-in (*P* < 0.001); ESI 2 (*P* < 0.01); ESI 3 (*P* < 0.001); and anticoagulation use (*P* < 0.05). Patients who presented to the tertiary academic ED had a significantly lower likelihood of unnecessary imaging (*P* < 0.01).

**Table 2.** The output of the logistic regression analysis shows that the variables significantly associated with overutilization of computed tomography C-spine include ED site, age, means of arrival, ESI score, and anticoagulation status.

Variable	Odds ratio	*P*-value	CI 2.5%	CI 97.5%
Academic ED	0.56	<0.05	0.39	0.79
Age 65–84	3.40	<0.001	1.93	6.09
Age 85+	3.63	<0.001	1.98	6.78
Walk-in arrival	2.61	<0.001	1.69	4.06
ESI 2	4.08	<0.01	1.70	11.46
ESI 3	5.35	<0.001	2.23	15.09
On anticoagulation	1.55	<0.05	1.01	2.40

*ED*, emergency department; *ESI*, Emergency Severity Index; *CI*, confidence interval.

## DISCUSSION

Our investigation identified multiple factors associated with overutilization of CT C-spine imaging. In addition, our study confirmed prior reports related to C-spine evaluation, redemonstrating the sensitivity of the NEXUS C-spine rule and the significant overutilization of cervical spine imaging. This study expands the very limited published literature on CT overutilization in the ED setting. It is the first multicenter investigation to evaluate CT C-spine overutilization and the first to study the topic in community EDs. These novel findings have potential implications for future efforts to reduce unnecessary CT imaging of the cervical spine.

The outcomes for NEXUS-negative patients in this study align with prior data validating the sensitivity of the NEXUS C-spine rule for identifying cervical spine fractures. None of the patients in the NEXUS-negative cohort were found to have a clinically significant cervical spine injury. Despite this sensitivity within our own patient population, nearly half of all cervical spine CTs were ordered despite the patient’s status as NEXUS negative.

Our regression analysis demonstrated that patient age was strongly associated with overutilization, with geriatric patients more likely to undergo CT imaging despite being NEXUS-negative. There are several factors that may be driving this association. Some physicians may be influenced in their practice by the Canadian C-spine rule, which recommends imaging for patients >65 years in age. Some clinicians may also be less judicious with imaging in geriatric patients, particularly because of a perceived higher likelihood of injury and lower long-term risk of radiation among older adults. Nonetheless, our results reinforce that age should not influence CT decision-making; none of the NEXUS-negative patients in our study population, including the patients who were over 65, were found to have a clinically significant fracture. Future efforts to reduce CT overutilization should emphasize that although radiation may be less of a concern in the elderly population, we did not find any additional risk based on age alone, and there are other significant negative ramifications of overutilization including cost, ED throughput, and incidental findings.

In contrast to age, concurrent CT head was surprisingly not associated with overutilization of CT C-spine. We hypothesize that some physicians may order a CT of the cervical spine on a NEXUS-negative patient if the patient is already going to be undergoing a CT head, and the clinician may not perceive much additional risk to ordering a concurrent CT C-spine. However, in this population, this variable was not significant, likely due to collinearity (though under the 70% threshold) between ordering of CT head and CT C-spine. The vast majority of the patients in this study underwent a concurrent CT head, and there was not a statistically significant difference between the NEXUS-positive and NEXUS-negative cohorts.

Means of arrival was also noted to be a significant variable in this model, with an increased likelihood of overutilization in patients who arrived as walk-ins. This is likely related to patient self-sorting of arrival modality. We hypothesized that patients who walk into the ED are categorically less likely to have a significant injury. Furthermore, walk-in patients are less likely to meet certain NEXUS criteria, such as having a distracting injury or a neurological deficit, which typically warrant EMS transport. Thus, it is likely that fewer CTs are ordered on walk-in patients, which may be driving this phenomenon, although a definitive explanation remains unclear.

Patients who were on anticoagulants were more likely to receive unnecessary CT imaging of the cervical spine. Our study was not designed to explore the underlying drivers of this behavior or to infer causality. It is possible that patients on anticoagulation were more likely to undergo CT head, even with relatively minor mechanisms of injury, and clinicians reflexively ordered a concurrent CT C-spine, as it has been established that a common practice pattern in our study population involves concurrent ordering of CT head and CT C-spine. However, further research would be needed to explore this hypothesis.

Additionally, ESI scores of 2 and 3 were also associated with overutilization. This finding was of unclear significance, but it may offer potential targets for future interventions to reduce CT overutilization. The ED site of presentation was significantly associated with overutilization in this study. At the academic tertiary care ED, there was significantly less overutilization compared to the community EDs. It is hypothesized that academic faculty and residents may be more likely to follow evidence-based guidelines than physicians practicing at community sites; however, there is notably significant overlap in the clinicians who practice at each of the sites in this study. This could also be a result of patient self-selection similar to mode of arrival discussed above. Further investigation is warranted with regard to our observations of overutilization in the academic center vs community sites because if this is a generalized phenomenon, it may better inform strategies to reduce CT overutilization on a national level. Furthermore, to date, findings related to resource utilization on teaching vs non-teaching settings have shown either equivalence or overuse in the academic setting.^17^^–^^20^ Our results appear to refute this trend, at least for CT C-spine utilization, for reasons that remain unclear.

The associations identified in this study can be used to inform strategies to improve dissemination and adoption of the NEXUS C-spine rule and, thus, reduce unnecessary CT imaging. A reduction in overutilization of CT imaging may reduce healthcare costs, minimize unnecessary radiation to patients, and improve ED throughput. At a minimum, emphasis on clinician education regarding sensitivity of NEXUS in elderly patients may be needed. It may also be prudent to focus educational strategies at community practice sites, which demonstrated higher rates of overutilization in our study. Additionally, this information could be used in audit and feedback strategies to include not just utilization rates but age ranges and rates of identifying significant injuries.

## LIMITATIONS

Foremost, our investigation was not designed to determine causality but rather only association. Therefore, while many of the variables used in this regression were correlated with overutilization of CT imaging, we were unable to prove a causative relationship for these factors. Furthermore, while our investigation did include four EDs that differed in size and patient populations, they were staffed by members of a single academic department of emergency medicine, limiting generalizability.

This study was also limited by the documentation that was available upon chart review. It is possible that NEXUS criteria could have been present but were not documented by the clinician, causing the rate of overutilization to be overestimated. However, given the clinical importance of each of the NEXUS criteria in the evaluation of patients with potential cervical spine injury, it is likely that positive criteria would have been included in the clinician’s documentation. In addition to reviewing clinician documentation, chart reviewers also reviewed lab studies, imaging, and nursing notes to identify any information regarding the NEXUS criteria.

To support feasibility, our study was limited to a two-month period. These occurred during winter months, and some of the patients had season-specific mechanisms of injury. For example, mechanisms of injury included skiing/snowboarding accidents and snowmobile collisions. Although none of the mechanisms of injury in this study were significantly associated with overutilization of CT imaging, there may be significant mechanisms of injury with seasonal variation that were not identified in this population due to the timing of the study.

Overall, the rate of overutilization at the sites in this study was higher than the rates identified previously in the literature. In this cohort, nearly one-half of the patients who underwent CT of the cervical spine were negative by NEXUS criteria. Although this is higher than previously reported rates, it should be noted that there is very limited published data on this topic, and prior research has been limited to academic medical centers.^12^^–^^14^ It is unclear whether or how this may have affected our observed results.

Lastly, there may be significant variability in individual physician practice patterns, which may be a factor associated with overutilization of CT imaging. However, due to the limited number of studies that were ordered by each physician during the study period, we were unable to assess the association between individual physician practice patterns and overutilization. Future investigation with a larger study population would be needed to answer this question.

## CONCLUSION

Computed tomography C-spine imaging continues to be overutilized in the ED setting. Factors that were associated with overuse of CT imaging include >65 years old, ESI scores 2 and 3, arrival by walk-in, anticoagulation, and presenting to a community ED. These findings may assist in guiding future interventions to optimize resource utilization and promote safer and more efficient emergency care.
